# ﻿A new species of the genus *Leptobrachella* (Amphibia, Anura, Megophryidae) from the Darongshan Nature Reserve, Guangxi, China

**DOI:** 10.3897/zookeys.1260.161514

**Published:** 2025-11-19

**Authors:** Wei-Cai Chen, Yong-Jian Bei, Peng Li

**Affiliations:** 1 Key Laboratory of Environment Change and Resources Use in Beibu Gulf Ministry of Education, Nanning Normal University, Nanning 530001, China Nanning Normal University Nanning China; 2 Guangxi Key Laboratory of Earth Surface Processes and Intelligent Simulation, Nanning Normal University, Nanning 530001, China Yulin Normal University Yulin China; 3 College of Biology and Pharmacy, Yulin Normal University, Yulin 537000, China Nanning Normal University Nanning China; 4 Key Laboratory of Mountain Biodiversity Conservation, Education Department of Guangxi Zhuang Autonomous Region, Yulin Normal University, Yulin 537000, China Yulin Normal University Yulin China

**Keywords:** Bioacoustics, morphology, phylogeny, sympatric distribution, taxonomy

## Abstract

A new species of the genus *Leptobrachella*, *L. darongshanensis***sp. nov.**, is described from the Darongshan Nature Reserve, Yulin City, Guangxi, China, integrating molecular, morphological, and bioacoustic evidence. The new species can be distinguished from its congeners by a combination of the following characters: (1) medium body size (SVL 24.9–27.7 mm in males; 32.0–35.4 mm in females); (2) dorsal skin rough with small, raised tubercles and ridges; (3) ventral surface creamy white with minute irregular textures and tiny pale brown spots laterally on the belly; (4) flanks bearing irregular black spots; (5) distinct black supratympanic line; (6) rudimentary toe webbing on toes I–IV and absence of lateral dermal fringes on toes; (7) distinct, continuous ventrolateral glandular line; (8) iris bicolored, upper half tangerine, lower half silver with black reticulations, pupil black with tangerine edges; (9) tibiotarsal articulation reaching the posterior margin of the eye when adpressed; (10) advertisement calls with dominant frequencies of 6.1–6.7 kHz at 20.0 °C. Phylogenetic analyses based on the mitochondrial 16S rRNA gene indicate that *L. darongshanensis***sp. nov.** and *L. shiwandashanensis* are sister taxa. The new species is currently known only from montane evergreen forests at elevations between 800 and 1,200 m within the Darongshan Nature Reserve, where it is sympatric with *L. yunkaiensis*.

## ﻿Introduction

The genus *Leptobrachella* Smith, 1925 is widely distributed across southern China, northeastern India, Myanmar, Thailand, Vietnam, Peninsular Malaysia, Borneo, and Natuna Island (Frost 2024). Currently comprising 110 species, 45 occur in China (AmphibiaChina 2025). Recent years have seen a surge in descriptions of new *Leptobrachella* species, indicating that the diversity of this genus remains underestimated. Most species occupy specialized microhabitats, such as headwater streams in montane forests. Their small body size and cryptic habits make them challenging to locate in the field.

The Darongshan Nature Reserve is located in Yulin City, Guangxi, China. It encompasses the Darongshan mountain range, with Lotus Peak reaching the highest elevation of 1,275.6 m. Situated on the southern edge of the South Subtropical Monsoon Climate Zone, the reserve is predominantly covered by monsoon evergreen broad-leaved forests. The annual average temperature ranges from 16.8 °C to 19.6 °C (July average: 26.1 °C). Annual precipitation is 1,800–2,200 mm, primarily between June and September, with an average annual relative humidity of 82.8% (Zheng and Qin 2022).

During amphibian surveys conducted from 2023 to 2025 within the Darongshan Nature Reserve, two morphotypes of *Leptobrachella* were encountered. No species of *Leptobrachella* had been previously reported from this reserve. Phylogenetic analysis using the mitochondrial 16S rRNA gene revealed that one morphotype (hereafter referred to as the putative new species) formed a sister clade to *L. shiwandashanensis* Chen, Peng, Pan, Liao, Liu & Huang, 2021, while the other morphotype clustered closely with *L. yunkaiensis* Wang, Li, Lyu & Wang, 2018. Morphological characterization and bioacoustic analyses demonstrated significant differences between the putative new species and *L. shiwandashanensis*, supporting its recognition as a distinct species. The second morphotype showed only minor morphological divergence from *L. yunkaiensis*, but due to the lack of vocal comparisons with topotypic *L. yunkaiensis*, we conservatively identify it as L. cf. yunkaiensis.

## ﻿Material and methods

### ﻿Sampling

**Figure 1.**
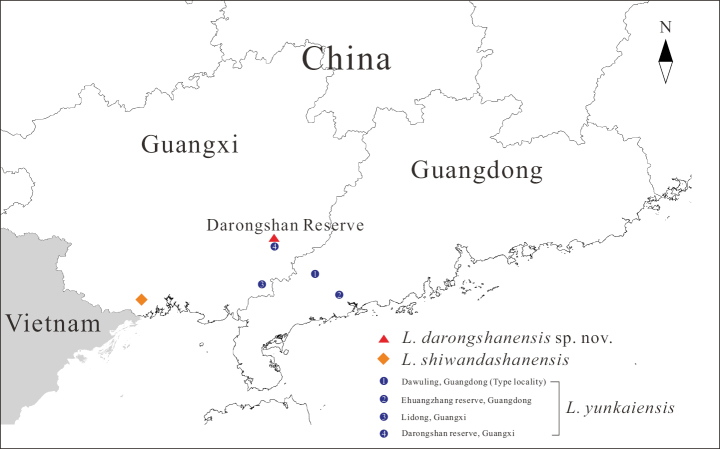
Localities of the new species, *L. shiwandashanensis*, and *L. yunkaiensis*.

Twenty-three adult specimens were collected within the Darongshan Nature Reserve, Yulin City, Guangxi, China (Fig. 1). Specimens were euthanized using isoflurane, fixed in 10% formalin, and subsequently stored in 75% ethanol as vouchers deposited at Nanning Normal University (NNU). Muscle tissue samples were preserved in 100% ethanol for molecular analysis.

### ﻿Morphological comparisons

Comparative morphological data were obtained from the literature (Suppl. material 2: table S1) and museum specimens (Suppl. material 2: table S2). Fourteen morphological characters were measured using digital calipers to the nearest 0.1 mm, following Rowley et al. (2016): snout-vent length (**SVL**), head length (**HL**, from tip of snout to posterior margin of jaw articulation), head width (**HW**, maximum width between left and right jaw articulations), snout length (**SNT**, from tip of snout to anterior corner of eye), eye diameter (**ED**, horizontal diameter of exposed eyeball), interorbital distance (**IOD**, shortest distance between anterior corners of orbits), horizontal tympanum diameter (**TD**), internarial distance (**IN**, minimum distance between inner margins of external nares), tibia length (**TIB**, from knee to tarsus), forelimb length (**FLL**, from elbow to tip of third finger), length of foot and tarsus (**TFL**, from tibiotarsal articulation to tip of toe IV), manus length (**ML**, from tip of third digit to proximal edge of inner palmar tubercle), hindlimb length (**HLL**, from tip of fourth toe to vent), and distance from proximal edge of femoral gland to knee (**FG-knee**). Sex was determined based on direct observation of calling behavior in the field or the presence of internal vocal sac openings.

### ﻿Molecular analyses

Genomic DNA was extracted from tissue samples using commercial kits (Tiangen Biotech Co. Ltd., Beijing, China). A fragment of the mitochondrial 16S rRNA gene was amplified and sequenced following Palumbi et al. (1991) using primers 16Sar_L (5’–CGCCTGTTTACCAAAAACAT–3’) and 16Sbr_H (5’–CCGGTCTGAACTCAG ATCACGT–3’). Polymerase chain reaction (PCR) conditions were: initial denaturation at 94 °C for 5 min; 35 cycles of denaturation at 94 °C for 35 s, annealing at 58 °C for 40 s, extension at 72 °C for 40 s; final extension at 72 °C for 10 min. PCR products were sequenced directly on an ABI Prism 3730 automated DNA sequencer (Applied Biosystems, USA). New sequences were deposited in GenBank (Accession nos. PX480157–PX480169, PX480170–PX480176).

Phylogenetic analyses employed Maximum Likelihood (ML) and Bayesian Inference (BI). The best-fit nucleotide substitution model (GTR+F+I) was selected using ModelFinder 2.2.0 (Kalyaanamoorthy et al. 2017) implemented in PhyloSuite v. 1.2.3 (Zhang et al. 2020), based on the Bayesian Information Criterion (BIC). ML analysis was performed in IQ-tree 2.2.2 (Nguyen et al. 2015) with 2000 ultrafast bootstrap replicates. BI was conducted using MrBayes v. 3.2 (Ronquist et al. 2012), running two independent analyses with four Markov chains each for 30 million generations, sampling every 1000 generations. The first 25% of sampled trees were discarded as burn-in. Uncorrected pairwise (*p*) distances were calculated in MEGA v. 11 (Tamura et al. 2021) using default settings.

### ﻿Bioacoustics analyses

Advertisement calls were recorded using a SONY PCM-A10 recorder. Ambient air temperature during recordings was 20.0 °C, measured with a Deli LE505 hand-held weather meter. Calls were analyzed and visualized using Raven Pro v. 1.6.1 (Cornell Laboratory of Ornithology, Ithaca, NY, USA). Spectrograms were generated using a Fast Fourier Transform (FFT) size of 512 points, 50% overlap, grid spacing of 172 Hz, and Hanning windows (Köhler et al. 2017).

## ﻿Results

### ﻿Molecular analyses

**Figure 2.**
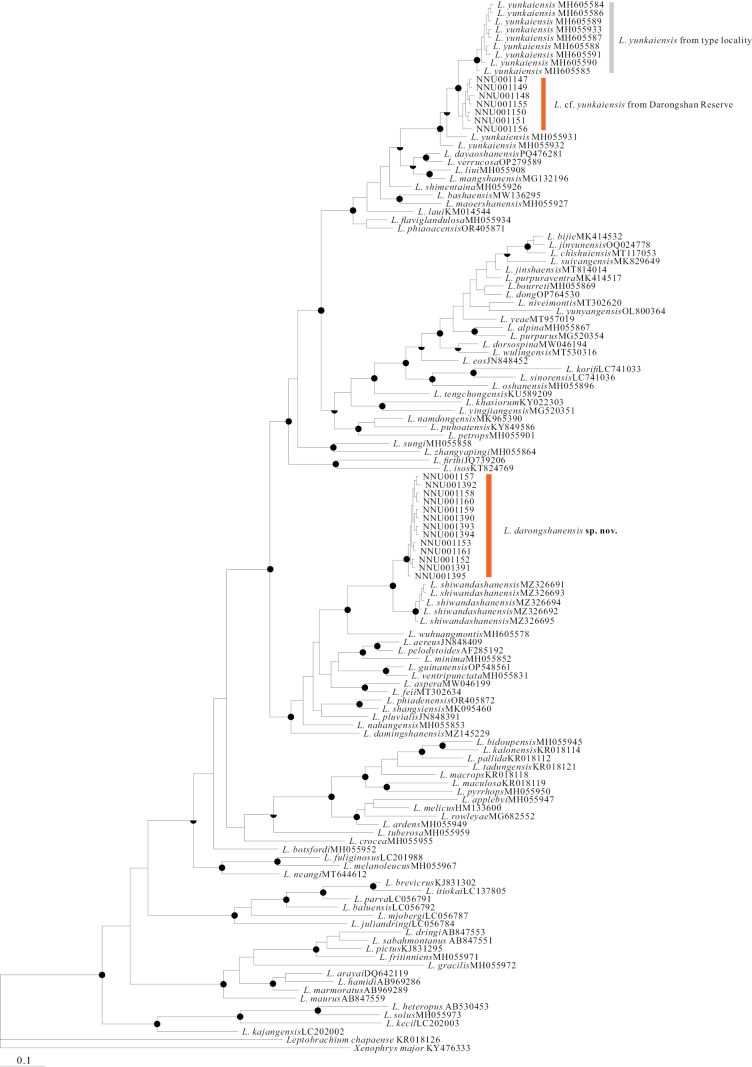
BI trees based on 16S rRNA fragments with Bayesian posterior probabilities/bootstrap supports on branches. Sold black means Bayesian posterior probabilities greater than 0.95 (upper half) and ultrafast bootstrap supports greater than 90 (lower half).

The final alignment consisted of 126 sequences of the 16S rRNA fragment (Table 1). Both ML and BI analyses yielded similar tree topologies (Fig. 2). Specimens collected from Darongshan formed two distinct monophyletic lineages. One lineage, *L. darongshanensis* sp. nov., was strongly supported (BPP = 1.00, UFB = 100) as the sister taxon to *L. shiwandashanensis*. The second lineage clustered within specimens identified as *L. yunkaiensis* from its type locality. Uncorrected *p*-distances between *L. darongshanensis* sp. nov. and *L. shiwandashanensis* ranged from 2.6% to 3.0%. Distances between the Darongshan L. cf. yunkaiensis specimens and topotypic *L. yunkaiensis* ranged from 1.4% to 2.7% (Suppl. material 2: table S3).

**Table 1.** DNA sequences used in this study. ‘*’ represents type locality.

ID	Species	Locality	Voucher no.	16S rRNA	Reference
1	*L. darongshanensis* **sp. nov.**	Darongshan Nature Reserve, Guangxi, China*	NNU001152	PX480157	This study
2	*L. darongshanensis* **sp. nov.**	Darongshan Nature Reserve, Guangxi, China*	NNU001153	PX480158	This study
3	*L. darongshanensis* **sp. nov.**	Darongshan Nature Reserve, Guangxi, China*	NNU001157	PX480159	This study
4	*L. darongshanensis* **sp. nov.**	Darongshan Nature Reserve, Guangxi, China*	NNU001158	PX480160	This study
5	*L. darongshanensis* **sp. nov.**	Darongshan Nature Reserve, Guangxi, China*	NNU001159	PX480161	This study
6	*L. darongshanensis* **sp. nov.**	Darongshan Nature Reserve, Guangxi, China*	NNU001160	PX480162	This study
7	*L. darongshanensis* **sp. nov.**	Darongshan Nature Reserve, Guangxi, China*	NNU001161	PX480163	This study
8	*L. darongshanensis* **sp. nov.**	Darongshan Nature Reserve, Guangxi, China*	NNU001390	PX480164	This study
9	*L. darongshanensis* **sp. nov.**	Darongshan Nature Reserve, Guangxi, China*	NNU001391	PX480165	This study
10	*L. darongshanensis* **sp. nov.**	Darongshan Nature Reserve, Guangxi, China*	NNU001392	PX480166	This study
11	*L. darongshanensis* **sp. nov.**	Darongshan Nature Reserve, Guangxi, China*	NNU001393	PX480167	This study
12	*L. darongshanensis* **sp. nov.**	Darongshan Nature Reserve, Guangxi, China*	NNU001394	PX480168	This study
13	*L. darongshanensis* **sp. nov.**	Darongshan Nature Reserve, Guangxi, China*	NNU001395	PX480169	This study
14	*L. shiwanshanensis*	Mt. Shiwandashan, Guangxi, China*	NNU202103213	MZ326692	Chen et al. 2021a
15	*L. shiwanshanensis*	Mt. Shiwandashan, Guangxi, China*	NNU202103214	MZ326693	Chen et al. 2021a
16	*L. shiwanshanensis*	Mt. Shiwandashan, Guangxi, China*	NNU202103215	MZ326694	Chen et al. 2021a
17	*L. shiwanshanensis*	Mt. Shiwandashan, Guangxi, China*	NNU202103146	MZ326691	Chen et al. 2021a
18	*L. shiwanshanensis*	Mt. Shiwandashan, Guangxi, China*	NNU202103261	MZ326695	Chen et al. 2021a
19	L. cf. yunkaiensis	Darongshan Nature Reserve, Guangxi, China	NNU001147	PX480170	This study
20	L. cf. yunkaiensis	Darongshan Nature Reserve, Guangxi, China	NNU001148	PX480171	This study
21	L. cf. yunkaiensis	Darongshan Nature Reserve, Guangxi, China	NNU001149	PX480172	This study
22	L. cf. yunkaiensis	Darongshan Nature Reserve, Guangxi, China	NNU001150	PX480173	This study
23	L. cf. yunkaiensis	Darongshan Nature Reserve, Guangxi, China	NNU001151	PX480174	This study
24	L. cf. yunkaiensis	Darongshan Nature Reserve, Guangxi, China	NNU001155	PX480175	This study
25	L. cf. yunkaiensis	Darongshan Nature Reserve, Guangxi, China	NNU001156	PX480176	This study
26	*L. yunkaiensis*	Dawuling, Guangdong, China*	SYS a004666	MH055933	Chen et al. 2018
27	*L. yunkaiensis*	Lidong, Guangxi, China	KIZ018211	MH055931	Chen et al. 2018
28	*L. yunkaiensis*	Ehuangzhang Nature Reserve, Guangdong, China	KIZ047782	MH055932	Chen et al. 2018
29	*L. yunkaiensis*	Dawuling, Guangdong, China*	SYS a004663	MH605584	Wang et al. 2018
30	*L. yunkaiensis*	Dawuling, Guangdong, China*	SYS a004664	MH605585	Wang et al. 2018
31	*L. yunkaiensis*	Dawuling, Guangdong, China*	SYS a004665	MH605586	Wang et al. 2018
32	*L. yunkaiensis*	Dawuling, Guangdong, China*	SYS a004666	MH605587	Wang et al. 2018
33	*L. yunkaiensis*	Dawuling, Guangdong, China*	SYS a004667	MH605588	Wang et al. 2018
34	*L. yunkaiensis*	Dawuling, Guangdong, China*	SYS a004668	MH605589	Wang et al. 2018
35	*L. yunkaiensis*	Dawuling, Guangdong, China*	SYS a004669	MH605590	Wang et al. 2018
36	*L. yunkaiensis*	Dawuling, Guangdong, China*	SYS a004690	MH605591	Wang et al. 2018
37	*L. aerea*	Quang Binh, Vietnam	ZFMK 86362	JN848409	Ohler et al. 2011
38	*L. alpina*	Caiyanghe, Yunnan, China	KIZ049024	MH055867	Chen et al. 2018
39	*L. applebyi*	Phong Dien Nature Reserve, Thua Thien-Hue, Vietnam	KIZ010701	MH055947	Chen et al. 2018
40	*L. arayai*	Borneo, Malaysia*	AE100/S9	DQ642119	Veith et al. 2006
41	*L. ardens*	Kon Ka Kinh National Park, Gia Lai, Vietnam*	ZMMU-NAP-06099	MH055949	Chen et al. 2018
42	*L. aspera*	Huanglianshan Nature Reserve, Lyuchun, Yunnan, China*	SYS a007743	MW046199	Wang et al. 2020
43	*L. baluensis*	Sabah, Borneo, Malaysia*	SP 21604	LC056792	Eto et al. 2015
44	*L. bashaensis*	Basha Nature Reserve, Guizhou, China*	GIB196404	MW136295	Lyu et al. 2020
45	*L. bidoupensis*	Bidoup-Nui Ba National Park, Lam Dong, Vietnam*	ZMMU-A-4797-01454	MH055945	Chen et al. 2018
46	*L. bijie*	Bijie City, Guizhou, China*	SYS a007313	MK414532	Wang et al. 2020
47	*L. botsfordi*	Lao Cai, Vietnam*	AMS R 176540	MH055952	Chen et al. 2018
48	*L. bourreti*	Mao’er Shan, Guangxi, China	KIZ019389	MH055869	Chen et al. 2018
49	*L. brevicrus*	Sarawak, Borneo, Malaysia*	ZMH A09365	KJ831302	Oberhummer et al. 2014
50	*L. chishuiensis*	Guizhou, China*	CIBCS20190518047	MT117053	Li et al. 2020
51	*L. crocea*	Thua Thien-Hue, Vietnam	ZMMU-NAP-02274	MH055955	Chen et al. 2018
52	*L. damingshanensis*	Wuming County, Guangxi, China*	NNU202103281	MZ145229	Chen et al. 2021b
53	*L. dayaoshanensis*	Dayaoshan, Jinxiu County, Guangxi, China*	NNU202103018	PQ476281	Yu et al. 2024
54	*L. dong*	Tongdao County, Hunan, China*	CIB SSC1757	OP764530	Liu et al. 2023
55	*L. dorsospina*	Yushe Forest Park, Shuicheng, Guizhou, China*	SYS a004961	MW046194	Wang et al. 2020
56	*L. dringi*	Borneo, Malaysia*	KUHE:55610	AB847553	Matsui et al. 2014a
57	*L. eos*	Phongsaly, Laos*	MNHN 2004.0274	JN848452	Ohler et al. 2011
58	*L. feii*	Yunnan, China*	KIZ048894	MT302634	Chen et al. 2020
59	*L. firthi*	Kon Tum, Vietnam*	AMS: R 176524	JQ739206	Rowley et al. 2012
60	*L. flaviglandulosa*	Xiaoqiaogou Nature Reserve, Yunnan, China*	KIZ016072	MH055934	Chen et al. 2018
61	*L. fritinniens*	Danum Valley Field Center, Sabah, Malaysia	FMNH 244800	MH055971	Chen et al. 2018
62	*L. fuliginosa*	Phetchaburi, Thailand	KUHE:20197	LC201988	Matsui et al. 2017
63	*L. gracilis*	Bukit Kana, Sarawak, Malaysia	FMNH 273682	MH055972	Chen et al. 2018
64	*L. guinanensis*	Shangsi County, Guangxi, China*	NNU00557	OP548561	Chen et al. 2024
65	*L. hamidi*	Borneo, Malaysia*	KUHE 17545	AB969286	Matsui et al. 2014b
66	*L. heteropus*	Peninsular, Malaysia	KUHE 15487	AB530453	Matsui et al. 2010
67	*L. isos*	Gia Lai, Vietnam*	AMS R 176480	KT824769	Rowley et al. 2015a
68	*L. itiokai*	Gunung Mulu National Park, Sarawak, Malaysia*	KUHE:55897	LC137805	Eto et al. 2016
69	*L. jinshaensis*	Lengshuihe Nature Reserve, Jinsha County, Guizhou, China*	CIBJS20200516001	MT814014	Cheng et al. 2021
70	*L. jinyunensis*	Mt. Jinyun, Beibei District, Chongqing, China*	CIB 119039	OQ024778	Shi et al. 2023
71	*L. juliandringi*	Sarawak, Borneo, Malaysia*	KUHE 17557	LC056784	Eto et al. 2015
72	*L. kajangensis*	Tioman, Malaysia*	LSUHC:4439	LC202002	Matsui et al. 2017
73	*L. kalonensis*	Binh Thuan, Vietnam*	IEBR A.2014.15	KR018114	Rowley et al. 2015b
74	*L. kecil*	Cameron, Malaysia *	KUHE:52439	LC202003	Matsui et al. 2017
75	*L. khasiorum*	Meghalaya, India*	SDBDU 2009.329	KY022303	Mahony et al. 2017
76	*L. korifi*	Doi Inthanon, Thailand*	KUHE 19134	LC741033	Matsui et al. 2023
77	*L. laui*	Wutongshan, Shenzhen city, China*	SYS a001507	KM014544	Sung et al. 2014
78	*L. liui*	Wuyishan, Fujian, China *	ZYCA907	MH055908	Chen et al. 2018
79	*L. macrops*	Dak Lak, Vietnam*	AMS R177663	KR018118	Rowley et al. 2015b
80	*L. maculosa*	Ninh Thuan, Vietnam*	AMS: R 177660	KR018119	Rowley et al. 2015b
81	*L. mangshanensis*	Mangshan, Hunan, China*	MSZTC201701	MG132196	Hou et al. 2018
82	*L. maoershanensis*	Mao’er Shan, Guangxi, China	KIZ07614	MH055927	Chen et al. 2018
83	*L. marmorata*	Borneo, Malaysia*	KUHE 53227	AB969289	Matsui et al. 2014b
84	*L. maura*	Borneo, Malaysia	SP 21450	AB847559	Matsui et al. 2014a
85	*L. melanoleuca*	Kapoe, Ranong, Thailand	KIZ018031	MH055967	Chen et al. 2018
86	*L. melica*	Ratanakiri, Cambodia*	MVZ 258198	HM133600	Rowley et al. 2010a
87	*L. minima*	Doi Phu Fa, Nan, Thailand	KIZ024317	MH055852	Chen et al. 2018
88	*L. mjobergi*	Sarawak, Borneo, Malaysia*	KUHE 47872	LC056787	Eto et al. 2015
89	*L. nahangensis*	Tuyen Quang, Vietnam*	ROM 7035	MH055853	Chen et al. 2018
90	*L. namdongensis*	Thanh Hoa, Vietnam*	VNUF A.2017.95	MK965390	Hoang Chen et al. 2019
91	*L. neangi*	Veal Veng District, Pursat, Cambodia*	CBC 1609	MT644612	Stuart and Rowley 2020
92	*L. niveimontis*	Yongde County, Yunnan, China *	KIZ028276	MT302620	Chen et al. 2020
93	*L. oshanensis*	Emei Shan, Sichuan, China*	Tissue ID: YPX37492	MH055896	Chen et al. 2018
94	*L. pallida*	Lam Dong, Vietnam*	UNS00510	KR018112	Rowley et al. 2015b
95	*L. parva*	Mulu National Park, Sarawak, Malaysia*	KUHE:55308	LC056791	Eto et al. 2015
96	*L. pelodytoides*	NA	TZ819	AF285192	Ziegler 2000
97	*L. petrops*	Ba Vi National Park, Ha Tay, Vietnam	ROM 13483	MH055901	Chen et al. 2018
98	*L. phiadenensis*	Phia Oac-Phia Den NP, Cao Bang Prov., Vietnam*	IEBR A.5205	OR405872	Luong et al. 2023
99	*L. phiaoacensis*	Phia Oac-Phia Den NP, Cao Bang Prov., Vietnam*	IEBR A. 5195	OR405871	Luong et al. 2023
100	*L. picta*	Borneo, Malaysia	UNIMAS 8705	KJ831295	Oberhummer et al. 2014
101	*L. pluvialis*	Lao Cai, Vietnam*	MNHN:1999.5675	JN848391	Ohler et al. 2011
102	*L. puhoatensis*	Nghe An, Vietnam*	VNMN 2016 A.22	KY849586	Rowley et al. 2017a
103	*L. purpurus*	Yunnan, China *	SYSa006530	MG520354	Yang et al. 2018
104	*L. purpuraventra*	Guizhou, China *	SYSa007281	MK414517	Wang et al. 2019
105	*L. pyrrhops*	Loc Bac, Lam Dong, Vietnam*	ZMMU-A-4873-00158	MH055950	Chen et al. 2018
106	*L. rowleyae*	Da Nang City, Vietnam*	ITBCZ2783	MG682552	Nguyen et al. 2018
107	*L. sabahmontanus*	Borneo, Malaysia*	BORNEENSIS 12632	AB847551	Matsui et al. 2014a
108	*L. shangsiensis*	Shangsi County, China*	NHMG1401032	MK095460	Chen et al. 2019
109	*L. shimentaina*	Shimentai Nature Reserve, Guangdong, China*	SYS a004712	MH055926	Chen et al. 2018
110	*L. sinorensis*	Mae Hong Son, Thailand*	KUHE 19816	LC741036	Matsui et al. 2023
111	*L. sola*	Gunung Stong, Kelantan, Malaysia	KU RMB20973	MH055973	Chen et al. 2018
112	*L. suiyangensis*	Guizhou, China *	GZNU20180606005	MK829649	Luo et al. 2020
113	*L. sungi*	Vinh Phuc, Vietnam *	ROM 20236	MH055858	Chen et al. 2018
114	*L. tadungensis*	Dak Nong, Vietnam*	UNS00515	KR018121	Rowley et al. 2015b
115	*L. tengchongensis*	Yunnan, China *	SYSa004598	KU589209	Yang et al. 2016
116	*L. tuberosa*	Kon Ka Kinh National Park, Gia Lai, Vietnam*	ZMMU-NAP-02275	MH055959	Chen et al. 2018
117	*L. ventripunctata*	Zhushihe, Yunnan, China *	SYSa004536	MH055831	Chen et al. 2018
118	*L. verrucosa*	Lianshan Bijiashan Nature Reserve, Guangdong, China	GEP a059	OP279589	Lin et al. 2022
119	*L. wuhuangmontis*	Pubei County, Guangxi, China *	SYS a003486	MH605578	Wang et al. 2018
120	*L. wulingensis*	Hunan, China *	CSUFT194	MT530316	Qian et al. 2020
121	*L. yeae*	Mount Emei, Sichuan, China *	CIBEMS20190422HLJ1-6	MT957019	Shi et al. 2021
122	*L. yingjiangensis*	Yunnan, China *	SYSa006532	MG520351	Yang et al. 2018
123	*L. yunyangensis*	Yunyang County, Chongqing, China *	GZNU20210622001	OL800364	Luo et al. 2022
124	*L. zhangyapingi*	Chiang Mai, Thailand *	KIZ07258	MH055864	Chen et al. 2018
125	*Xenophrys major*	Kon Tum Province, Vietnam	AMS R173870	KY476333	Rowley et al. 2017b
126	*Leptobrachium chapaense*	Lao Cai Province, Vietnam	AMS R 171623	KR018126	Rowley et al. 2015b

### ﻿Bioacoustics

**Figure 3.**
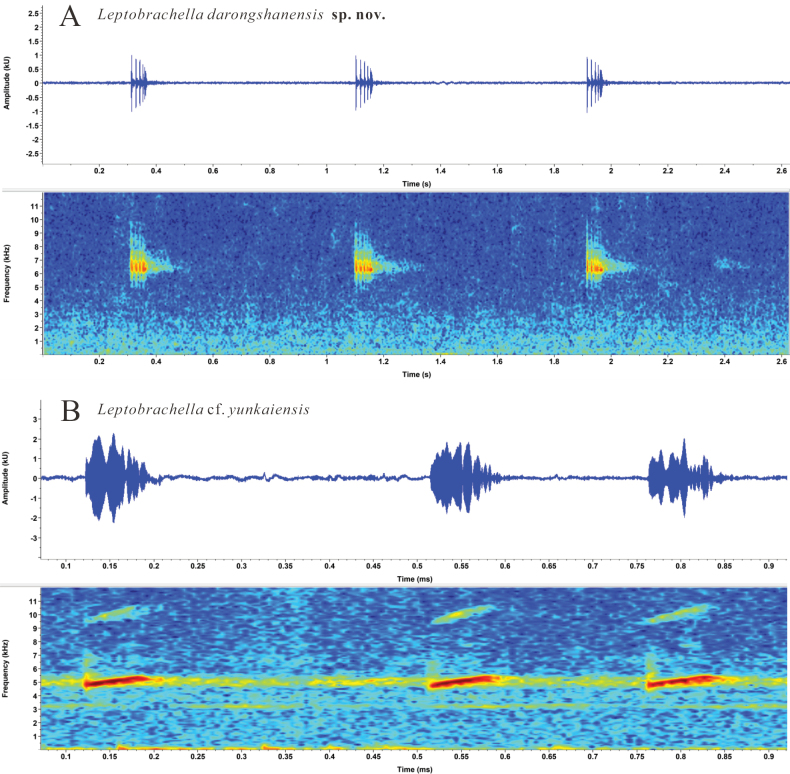
Advertisement call spectrograms of *L. darongshanensis* sp. nov. and L. cf. yunkaiensis.

Advertisement calls of three individuals of *L. darongshanensis* sp. nov. and three individuals of L. cf. yunkaiensis were recorded at approximately 20.0 °C. The calls of *L. darongshanensis* sp. nov. differed significantly from those of the sympatric L. cf. yunkaiensis in call duration (59.1–109.2 ms, mean ± SD: 77.9 ± 14.5 ms, *n* = 75 calls vs 25.8–46.6 ms, 35.6 ± 6.2 ms, *n* = 23), call interval (334.8–763.7 ms, 521.2 ± 106.7 ms, *n* = 74 intervals vs 150.1–559.5 ms, 309.5 ± 132.5 ms, *n* = 23), and dominant frequency (6.1–6.7 kHz vs 4.9–5.3 kHz). The calls of *L. darongshanensis* sp. nov. also differed markedly from its sister species, *L. shiwandashanensis*, in call duration (59.1–109.2 ms, 77.9 ± 14.5 ms, *n* = 75 vs 194.0–277.0 ms, 226.6 ± 16.4 ms, *n* = 80), call interval (334.8–763.7 ms, 521.2 ± 106.7 ms, *n* = 74 vs 134.0–186 ms, mean: 153.1 ms, *n* = 23), and dominant frequency (6.1–6.7 kHz vs 5.3–5.7 kHz) (Table 2, Fig. 3).

**Table 2.** Comparisons of characters of advertisement calls of the new species, *L. shiwandashanensis*, and L. cf. yunkaiensis.

Species	Dominant frequency (kHz)	Call duration (ms)	Call interval (ms)	Temperature (°C)	References
*L. darongshanensis* sp. nov.	6.1–6.7	77.9 (59.1–109.2)	521.2 (334.8–763.7)	20.0	This study
*L. shiwandashanensis*	5.3–5.7	226.6 (194.0–277.0)	153.1 (134.0–186.0)	23.0	Chen et al. 2021a
L. cf. yunkaiensis	4.9–5.3	35.6 (25.8–46.6)	309.5 (150.1–559.5)	20.0	This study

### ﻿Morphology

Phylogenetic results indicated *L. darongshanensis* sp. nov. is closely related to *L. shiwandashanensis*. However, it differs from *L. shiwandashanensis* in several morphological characters, including iris coloration, relative eye diameter, supratympanic line color, toe webbing development, and ventrolateral glandular line continuity (see Comparisons below). Specimens of the second lineage (L. cf. yunkaiensis) were morphologically highly similar to topotypic *L. yunkaiensis* (see Taxonomic account for *L. yunkaiensis*).

Based on the congruent results from molecular phylogenetics, bioacoustics, and morphology, we describe the first lineage as a new species, *L. darongshanensis* sp. nov. The second lineage is identified as L. cf. yunkaiensis, representing a new population record.

### ﻿Taxonomic account

#### ﻿ Leptobrachella darongshanensis sp. nov.

Taxon classificationAnimaliaAnuraMegophryidae

E7C60536-0146-577F-82EF-8C054A7497E5

https://zoobank.org/9F3C1115-FFF1-4D04-A88C-5939173E1F40

Fig. 4A–F

##### Type material.

***Holotype*** • NNU 001390, adult male, collected at Darongshan Nature Reserve, Yulin City, Guangxi, China (22.868°N, 110.202°E; elevation 1048 m) by Wei-Cai Chen on 19 March 2025.

***Paratypes*** • NNU 001152–001153 (two adult males), NNU 001157–001161 (four adult females), collected at the same locality as the holotype on 17 May 2023 by Wei-Cai Chen and Yong-Jian Bei. • NNU 001391–001393 (three adult males), NNU 001394–001397 (four adult females), collected at the same locality as the holotype on 19 March 2025 by Wei-Cai Chen.

##### Diagnosis.

*Leptobrachella darongshanensis* sp. nov. is assigned to the genus *Leptobrachella* based on molecular phylogenetic results and the following generic morphological characters: small size, presence of an inner metacarpal tubercle, absence of vomerine teeth, distinct tympanum, and the presence of macro-glands (pectoral and femoral glands, often including supra-axillary and ventrolateral glands). *Leptobrachella darongshanensis* sp. nov. can be distinguished from its congeners by the following combination of characters: (1) medium body size (SVL 24.9–27.7 mm in males; 32.0–35.4 mm in females); (2) dorsal skin rough with small, raised tubercles and ridges; (3) ventral surface creamy white with minute irregular textures and tiny pale brown spots laterally on the belly; (4) flanks bearing irregular black spots; (5) distinct black supratympanic line extending from posterior corner of eye to supra-axillary gland; (6) rudimentary toe webbing present between toes I–IV, lateral dermal fringes absent on toes; (7) ventrolateral glandular line distinct and continuous; (8) iris bicolored, upper half tangerine, lower half silver with black reticulations; pupil black with tangerine edges; (9) tibiotarsal articulation reaching the posterior margin of the eye when hindlimb is adpressed along body; (10) advertisement call dominant frequency 6.1–6.7 kHz at 20.0 °C.

##### Description of holotype.

Adult male (SVL 27.7 mm). Head width subequal to length (HW/HL = 0.98). Snout projecting, extending beyond lower jaw; nostril oval, closer to snout tip than to eye; canthus rostralis rounded; loreal region concave, sloping towards lips; interorbital region flat; pupil vertical; eye diameter larger than snout length (ED/SNT = 1.16); internarial distance subequal to interorbital distance (IN/IOD = 0.98); tympanum distinct, round, concave, diameter much smaller than eye diameter (TD/ED = 0.44); distinct, raised supratympanic fold extending from posterior corner of eye to supra-axillary gland. Vomerine teeth absent. Tongue pyriform, notched posteriorly.

**Figure 4.**
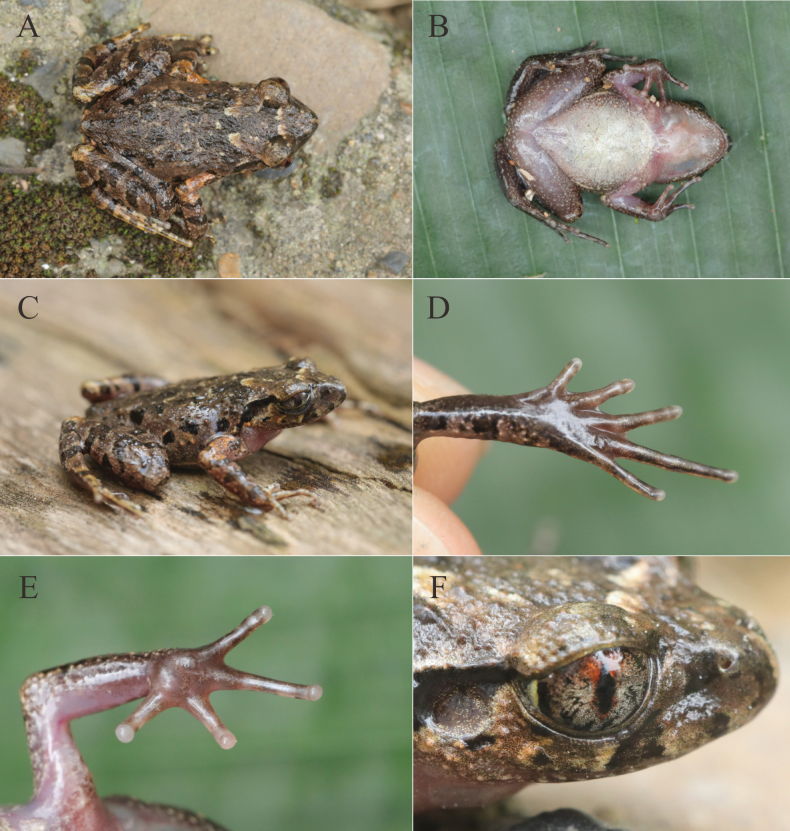
The holotype of *L. darongshanensis* sp. nov. **A.** Dorsal view; **B.** Ventral view; **C.** Dorsolateral view; **D.** Ventral view of foot; **E.** Ventral view of hand; **F.** Color pattern of eye.

Fingers lacking webbing and lateral fringes; tips slightly swollen; relative finger lengths I < II < IV < III; nuptial pads absent; subarticular tubercles absent; inner palmar tubercle prominent, oval; outer palmar tubercle prominent, rounded; inner and outer palmar tubercles separated. Toes lacking lateral fringes, tips rounded, not swollen; relative toe lengths I < II < V < III < IV; subarticular tubercles absent, replaced by low, indistinct dermal ridges; inner metatarsal tubercle large, elongated; outer metatarsal tubercle absent; webbing rudimentary between toes I–IV. Tibia length ~ 47% of SVL (TIB/SVL = 0.47); tibiotarsal articulation reaching posterior margin of eye when hindlimb is adpressed anteriad; heels not meeting when thighs are held at right angles to body axis (Fig. 4A–F).

Dorsal skin texture rough with numerous small, raised tubercles and short ridges; ventral skin smooth; pectoral glands oval, creamy white, ~1.4 mm in diameter; femoral glands oval, ~1.1 mm in diameter, positioned closer to knee than to vent; supra-axillary glands small, round, ~1.0 mm in diameter; ventrolateral glandular line distinct and continuous; limbs with sparse tubercles; a pair of small tubercles present above the cloaca (Fig. 4A–F).

***Color of holotype in life*.** Dorsum brown with distinct chocolate markings, including a dark brown triangular marking between eyes and a dark brown ‘W’-shaped marking on the scapular region, interspersed with irregular dark spots; tympanum pale brown; distinct black supratympanic line from posterior corner of eye to supra-axillary gland; three dark brown spots on upper lip; flanks with five irregular black spots and numerous small creamy yellow tubercles; dorsal surfaces of hindlimbs with three distinct transverse dark brown bars; dorsal surfaces of forelimbs with three transverse dark brown bars; elbows, upper arms, and tibiotarsal regions pale yellow; ventral surface creamy white with minute irregular textures and tiny pale brown spots laterally on belly; throat and chest pale pinkish; pectoral and femoral glands creamy white; supra-axillary glands pale yellow; iris bicolored, upper half tangerine, lower half silver with black reticulations; pupil black, surrounded by a tangerine ring (Fig. 4A–F).

***Color of holotype in preservative*.** Dorsum dark brown; transverse dark bars on limbs distinct; ventral surfaces creamy white; pectoral, femoral, supra-axillary, and ventrolateral glands creamy white.

**Figure 5.**
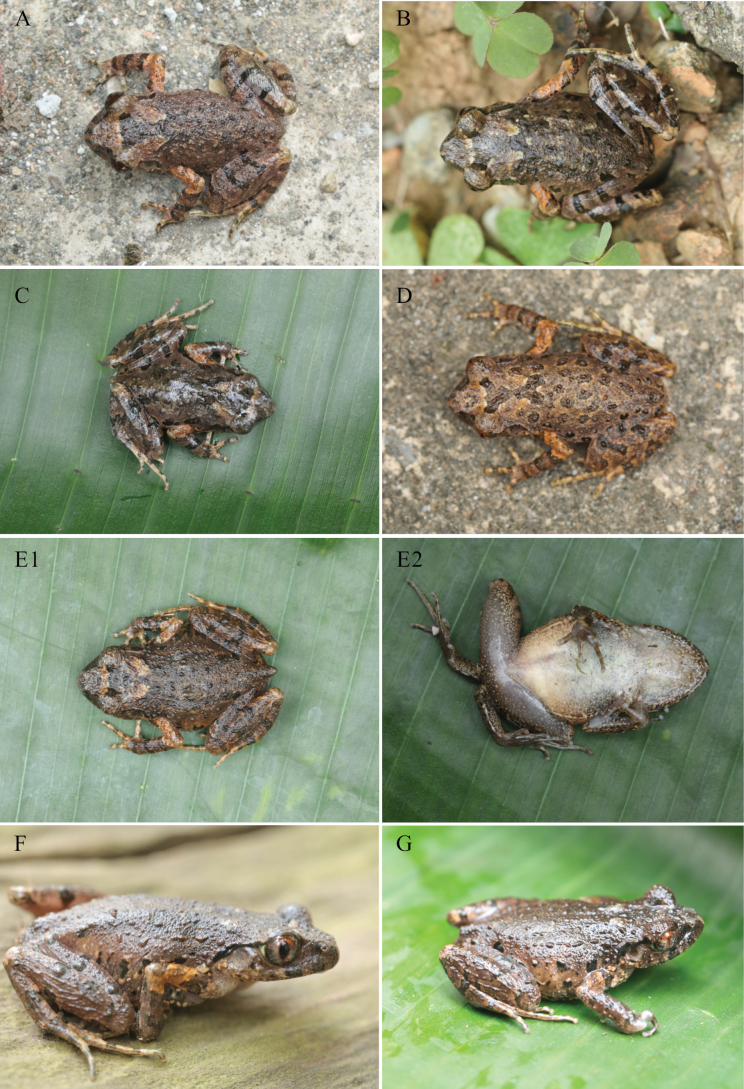
Variation of *L. darongshanensis* sp. nov. **A.**NNU 001391; **B.**NNU 001392; **C.**NNU 001393; **D.**NNU 001394; **E1.**NNU 001395 (dorsal view); **E2.**NNU 001395 (ventral view); **F.**NNU 001396; **G.**NNU 001397.

***Variation*.** Measurements of the type series are presented in Table 3. Coloration and pattern in paratypes are generally consistent with the holotype (Fig. 5). Variation is observed primarily in the intensity and distribution of dark dorsal spots and flank spots. For example, specimen NNU001394 exhibits more pronounced brown dorsal spotting (Fig. 5D).

**Table 3.** Measurements of *Leptobrachella darongshanensis* sp. nov.

	Males		Female	
	Ranges (mm) (*n* = 6)	Mean ± SD (mm)	Ranges (mm) (*n* = 9)	Mean ± SD (mm)
SVL	24.9–27.7	26.6 ± 1.0	32.0–35.4	33.8 ± 1.0
HL	9.5–11.1	10.0 ± 0.7	11.3–12.6	12.0 ± 0.5
HW	9.0–9.9	9.9 ± 0.2	10.8–11.7	11.4 ± 0.3
SNT	3.3–3.8	3.5 ± 0.2	3.3–4.9	4.3 ± 0.5
ED	3.6–4.0	3.8 ± 0.2	4.0–4.8	4.5 ± 0.3
IOD	2.3–2.9	2.7 ± 0.2	3.0–3.6	3.3 ± 0.2
TD	1.6–2.0	1.8 ± 0.1	1.8–2.4	2.1 ± 0.2
IN	2.5–2.8	2.6 ± 0.1	2.9–3.6	3.2 ± 0.2
TIB	12.1–12.9	12.5 ± 0.4	14.5–15.4	15.0 ± 0.3
FLL	12.6–13.6	13.2 ± 0.4	14.9–16.5	15.7 ± 0.5
TFL	17.6–19.1	18.3 ± 0.6	20.2–21.9	21.2 ± 0.6
ML	6.5–7.3	6.9 ± 0.3	7.6–8.6	7.9 ± 0.3
HLL	36.4–39.1	38.1 ± 1.0	45.2–48.7	46.5 ± 1.3
FG-knee	4.1–4.7	4.4 ± 0.2	4.4–6.1	5.4 ± 0.5

***Measurements of holotype*** (in mm): SVL 27.7, HL 9.5, HW 9.3, SNT 3.4, ED 4.0, IOD 2.6, TD 1.8, IN 2.5, TIB 12.9, FLL 13.5, TFL 19.1, ML 7.1, HLL 39.1, FG-knee 4.1.

##### Ecology and distribution.

*Leptobrachella darongshanensis* sp. nov. is currently known only from montane evergreen broad-leaved forest within the Darongshan Nature Reserve, Yulin City, Guangxi, China, at elevations between 800 and 1,200 m a.s.l. Individuals were found calling near rocky streams from March to May. All females collected in March were gravid (Fig. 5E–G). The new species occurs syntopically with *L. yunkaiensis* along the same stream sections.

##### Etymology.

The specific epithet *darongshanensis* is a toponym derived from the type locality, Mt. Darongshan. The suggested English common name is Darongshan Leaf Litter Toad. The suggested Chinese common name is Da Rong Shan Zhang Tu Chan (大容山掌突蟾).

##### Comparisons.

Comparisons focus on phylogenetically close relatives (Fig. 2) and the sympatric species *L. yunkaiensis*. *Leptobrachella darongshanensis* sp. nov. differs from its sister species *L. shiwandashanensis* by: larger relative eye diameter (ED/SNT = 1.16 vs 0.92); black (vs grey) supratympanic line; rudimentary toe webbing on toes I–IV (vs webbing absent); continuous (vs interrupted) ventrolateral glandular line; pupil ringed with tangerine (vs no distinct colored ring); iris bicolored, upper half tangerine, lower half silver with black reticulations (vs upper half brownish-red, lower half silver). Significant bioacoustic differences exist: shorter call duration (77.9 ± 14.5 ms vs 226.6 ± 16.4 ms), longer call interval (521.2 ± 106.7 ms vs mean 153.1 ms), and higher dominant frequency (6.1–6.7 kHz vs 5.3–5.7 kHz) (Table 2, Fig. 3).

*Leptobrachella darongshanensis* sp. nov. differs from *L. aerea* (Rowley, Stuart, Richards, Phimmachak & Sivongxay, 2010) by: absence of lateral dermal fringes on toes (vs narrow fringes present); relative finger lengths I < II < IV < III (vs I < II = IV < III); tibiotarsal articulation reaching posterior eye margin (vs reaching snout tip) when leg adpressed.

*Leptobrachella darongshanensis* sp. nov. differs from *L. aspera* Wang, Lyu, Qi & Wang, 2020 by: larger body size in males (SVL 24.9–27.7 mm vs 22.4 mm in single male) and females (32.0–35.4 mm vs 25.0–26.4 mm); iris bicolored, upper tangerine, lower silver with black reticulations (vs upper amber, lower silver); absence of lateral dermal fringes on toes (vs narrow fringes present); venter creamy white with minute textures (vs creamy white with distinct regular dark patches).

*Leptobrachella darongshanensis* sp. nov. differs from *L. damingshanensis* Chen, Yu, Cheng, Meng, Wei, Zhou & Lu, 2021 and *L. nahangensis* (Lathrop, Murphy, Orlov & Ho, 1998) by: significantly smaller male body size (SVL 24.9–27.7 mm vs 33.6–34.4 mm in *L. damingshanensis*, 40.8 mm in *L. nahangensis*).

*Leptobrachella darongshanensis* sp. nov. differs from *L. feii* Chen, Yuan & Che, 2020 by: larger body size in males (SVL 24.9–27.7 mm vs 21.5–22.7 mm) and females (32.0–35.4 mm vs 25.7 mm in single female); venter creamy white with minute textures (vs distinct black blotches on chest and belly); absence of lateral dermal fringes on toes (vs narrow fringes present).

*Leptobrachella darongshanensis* sp. nov. differs from *L. guinanensis* Chen, Li, Peng & Liu, 2024 by: smaller body size in males (SVL 24.9–27.7 mm vs 30.5–32.5 mm) and females (32.0–35.4 mm vs 38.7–41.8 mm); absence of lateral dermal fringes on toes (vs wide fringes present); rudimentary toe webbing (vs webbing basal, ~1/3 webbed); iris bicolored, upper tangerine, lower silver with black reticulations (vs upper pale copper, lower silver).

*Leptobrachella darongshanensis* sp. nov. differs from *L. minima* (Taylor, 1962) by: rough dorsal skin with tubercles and ridges (vs dorsum smooth); iris bicolored, upper tangerine, lower silver with black reticulations (vs upper dark gold, lower gray).

*Leptobrachella darongshanensis* sp. nov. differs from *L. pelodytoides* (Boulenger, 1893) by: absence of lateral dermal fringes on toes (vs narrow fringes present); rudimentary toe webbing (vs extensive webbing).

*Leptobrachella darongshanensis* sp. nov. differs from *L. phiadenensis* Luong, Hoang, Pham, Ziegler & Nguyen, 2023 by: absence of lateral dermal fringes on toes (vs narrow fringes present); rudimentary toe webbing (vs webbing absent); black supratympanic line (vs orange); venter creamy white with minute textures (vs white with dark speckling on outer margins).

*Leptobrachella darongshanensis* sp. nov. differs from *L. pluvialis* (Ohler, Marquis, Swan & Grosjean, 2000) by: larger male body size (SVL 24.9–27.7 mm vs 21.3–22.3 mm); creamy white venter with minute textures (vs pigmented venter); tibiotarsal articulation reaching posterior eye margin (vs reaching nostril) when leg adpressed.

*Leptobrachella darongshanensis* sp. nov. differs from *L. shangsiensis* Chen, Liao, Zhou & Mo, 2019 by: absence of lateral dermal fringes on toes (vs narrow fringes present); larger relative eye diameter (ED/SNT = 1.16 vs 0.78); continuous ventrolateral glandular line (vs discrete glands).

*Leptobrachella darongshanensis* sp. nov. differs from *L. ventripunctata* (Fei, Ye & Li, 1990) by: creamy white venter with minute textures and tiny lateral spots (vs belly creamy white with numerous scattered brown spots); rudimentary toe webbing on toes I–IV (vs webbing absent).

*Leptobrachella darongshanensis* sp. nov. differs from *L. wuhuangmontis* Wang, Yang & Wang, 2018 by: iris bicolored, upper tangerine, lower silver with black reticulations (vs upper coppery yellow, lower silver); venter creamy white with minute textures and tiny lateral pale brown spots (vs greyish white mixed with tiny white and black dots); rudimentary toe webbing and absence of lateral fringes on toes (vs toes with narrow lateral fringes and rudimentary webbing).

*Leptobrachella darongshanensis* sp. nov. occurs syntopically with *L. yunkaiensis*. The new species differs from *L. yunkaiensis* by: creamy white venter with minute textures and tiny lateral pale brown spots (vs belly pinkish with distinct or indistinct pale/dark brown speckling); absence of lateral dermal fringes on fingers (vs distinct fringes present); absence of lateral dermal fringes on toes (vs wide fringes present).

*Leptobrachella darongshanensis* sp. nov. differs from congeners occurring south of the Isthmus of Kra (e.g., *L. arayai* (Matsui, 1997), *L. dringi* (Dubois, 1987), *L. fritinniens* (Dehling & Matsui, 2013), *L. gracilis* (Günther, 1872), *L. hamidi* (Matsui, 1997), *L. heteropus* (Boulenger, 1900), *L. kajangensis* (Grismer, Grismer & Youmans, 2004), *L. kecil* (Matsui, Belabut, Ahmad & Yong, 2009), *L. marmorata* (Matsui, Zainudin & Nishikawa, 2014), *L. maura* (Inger, Lakim, Biun & Yambun, 1997), *L. melanoleuca* (Matsui, 2006), *L. picta* Malkmus, 1992, *L. platycephala* (Dehling, 2012), *L. sabahmontana* (Matsui, Nishikawa & Yambun, 2014), and *L. sola* Matsui, 2006) by the presence of supra-axillary glands and a distinct ventrolateral glandular line, which are absent in these species.

Furthermore, *L. darongshanensis* sp. nov. has a larger body size (male SVL 24.9–27.7 mm, female 32.0–35.4 mm) compared to: *L. baluensis* Smith, 1931 (male SVL 14.9–15.9 mm), *L. bondangensis* Eto, Matsui, Hamidy, Munir & Iskandar, 2018 (male SVL 17.8 mm), *L. brevicrus* Dring, 1983 (male SVL 17.1–17.8 mm), *L. fusca* Eto, Matsui, Hamidy, Munir & Iskandar, 2018 (male SVL 16.3 mm), *L. itiokai* Eto, Matsui & Nishikawa, 2016 (male SVL 15.2–16.7 mm), *L. juliandringi* Eto, Matsui & Nishikawa, 2015 (male SVL 17.0–17.2 mm), *L. mjobergi* Smith, 1925 (male SVL 15.7–19.0 mm), *L. natunae* (Günther, 1895) (male SVL 17.6 mm), *L. palmata* Inger & Stuebing, 1992 (male SVL 14.4–16.8 mm), *L. parva* Dring, 1983 (male SVL 15.0–16.9 mm), and *L. serasanae* Dring, 1983 (female SVL 16.9 mm). It is smaller than *L. dushanensis* Li, Li, Cheng, Liu, Wei & Wang, 2024 (male SVL 31.9–32.9 mm), *L. sungi* (Lathrop, Murphy, Orlov & Ho, 1998) (male SVL 48.3–52.7 mm), and *L. zhangyapingi* (Jiang, Yan, Suwannapoom, Chomdej & Che, 2013) (male SVL 45.8–52.5 mm). The presence of black spots on the flanks distinguishes it from species lacking such spots, including *L. aerea*, *L. botsfordi* (Rowley, Dau & Nguyen, 2013), *L. crocea* (Rowley, Hoang, Le, Dau & Cao, 2010), *L. eos* (Ohler, Wollenberg, Grosjean, Hendrix, Vences, Ziegler & Dubois, 2011), *L. firthi* (Rowley, Hoang, Le, Dau & Cao, 2012), *L. graminicola* Nguyen, Tapley, Nguyen, Luong & Rowley, 2021, *L. isos* (Rowley, Stuart, Neang, Hoang, Dau, Nguyen & Emmett, 2015), *L. pallida* (Rowley, Tran, Le, Dau, Peloso, Nguyen, Hoang, Nguyen & Ziegler, 2016), *L. petrops* (Rowley, Dau, Hoang, Le, Cutajar & Nguyen, 2017), and *L. tuberosa* (Inger, Orlov & Darevsky, 1999). Finally, the dominant frequency of its advertisement call (6.1–6.7 kHz) differs from that reported for other species (Suppl. material 2: table S4).

#### ﻿ Leptobrachella yunkaiensis

Taxon classificationAnimaliaAnuraMegophryidae

Wang, Li, Lyu & Wang, 2018

58F36D1B-57E6-510D-A311-50A686F2E9DC

**Figure 6.**
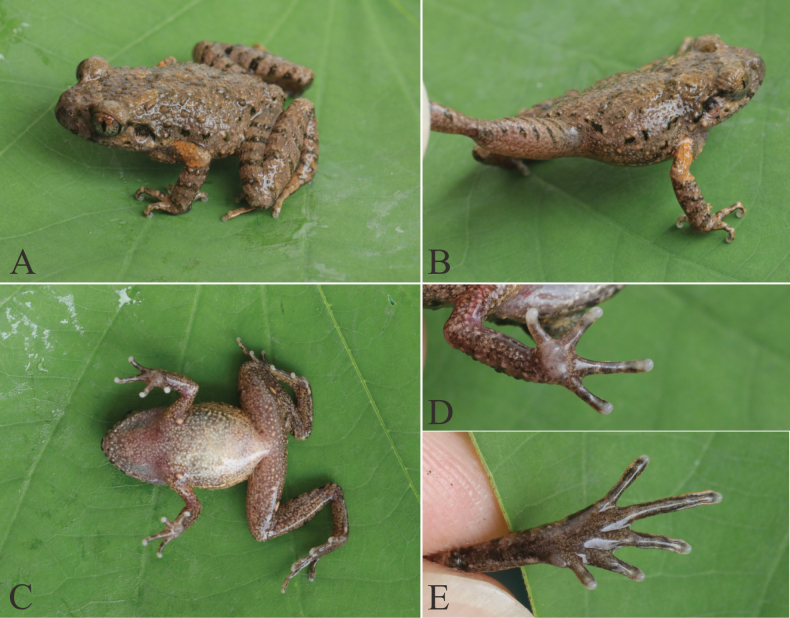
Morphological characters of L. cf. yunkaiensis from Guangxi (adult male, voucher no. NNU 001147). **A.** Dorsal view; **B.** Dorsolateral view; **C.** Ventral view; **D.** Ventral view of hand; **E.** Ventral view of foot.

Fig. 6, Suppl. material 1

##### Specimens examined.

• Seven adult males: NNU 001147–NNU 001151, NNU 001154, NNU 001155; • one adult female: NNU 001156, collected from the Darongshan Nature Reserve, Yulin City, Guangxi, China (22.860°N, 110.208°E; elevation 1125 m) by Wei-Cai Chen on 19 March 2023.

##### Description of Guangxi population.

The morphological characters of the Darongshan specimens align closely with the original description of *L. yunkaiensis* by Wang et al. (2018). Measurements are summarized in Table 4. Males SVL 24.6–27.2 mm; head length subequal to width (HL 9.3–10.5 mm; HW 9.4–10.5 mm); snout projecting beyond lower jaw; nostril oval, closer to snout tip than to eye; canthus rostralis rounded; loreal region concave, sloping; interorbital region flat; pupil vertical; ED 3.5–4.2 mm; IN 2.5–3.1 mm; tympanum distinct, round, concave; distinct, raised supratympanic fold from posterior corner of eye to supra-axillary gland; vomerine teeth absent; tongue pyriform, notched posteriorly.

**Table 4.** Measurements of L. cf. yunkaiensis from the Darongshan Nature Reserve, Yulin City, Guangxi, China.

	Males		Female
	Ranges (mm) (*n* = 7)	Mean ± SD (mm)	*n* = 1
SVL	24.6–27.2	25.8 ± 0.9	30.1
HL	9.3–10.5	9.9 ± 0.4	11.3
HW	9.4–10.5	9.8 ± 0.4	10.7
SNT	3.4–4.2	3.9 ± 0.3	4.0
ED	3.5–4.2	3.8 ± 0.2	4.2
IOD	2.7–3.2	2.8 ± 0.2	3.3
TD	1.7–2.1	1.9 ± 0.2	2.1
IN	2.5–3.1	2.8 ± 0.2	3.2
TIB	12.3–13.8	13.0 ± 0.6	13.7
FLL	11.9–13.7	12.8 ± 0.6	13.8
TFL	16.8–19.0	18.2 ± 0.8	19.2
ML	6.6–7.4	7.0 ± 0.3	6.8
HLL	36.3–40.7	38.9 ± 1.7	40.2
FG-knee	3.9–5.3	4.6 ± 0.4	5.1

***Fingers*** lacking webbing and fringes; tips slightly swollen; relative finger lengths I < II ≈ IV < III; nuptial pads absent; subarticular tubercles absent; inner and outer palmar tubercles prominent, separated. Toes lacking webbing except rudimentary between II and III; lateral dermal fringes narrow to wide; tips oval, not swollen; relative toe lengths I < II < V < III < IV; subarticular tubercles absent, replaced by indistinct dermal ridges; inner metatarsal tubercle elongated; outer metatarsal tubercle absent; tibiotarsal articulation reaching mid-eye level when leg adpressed; heels meeting when thighs held at right angles to body.

***Dorsal skin*** rough with small tubercles; ventral skin smooth; pectoral glands rounded, creamy white, ~1.3 mm diameter; femoral glands oval, ~1.6 mm diameter, closer to knee than vent; supra-axillary glands small, round, ~0.5 mm diameter; ventrolateral glandular line distinct and continuous; limbs with sparse tubercles.

***Dorsum*** brown with sparse pale yellow markings; dark brown interorbital triangle; tympanum brown; brown supratympanic line; dark brown spots on upper lip; flanks with irregular black spots and creamy yellow tubercles; dorsal thigh with 3–5 distinct transverse dark brown bars; dorsal forearms with three distinct transverse dark brown bars; elbows and upper arms tangerine; ventral surface pink or creamy white with minute irregular creamy textures; ventral limbs with tiny irregular creamy yellow spots; pectoral, femoral, supra-axillary glands creamy yellow; pupil black; iris bicolored, upper half tangerine, lower half silver (Fig. 6, Suppl. material 1).

##### Distribution.

The type locality of *L. yunkaiensis* is Dawuling Forest Station, Maoming City, Guangdong, China (Wang et al. 2018). Previous records include Ehuangzhang Nature Reserve, Guangdong and Lidong, Bobai City, Guangxi, China (Chen et al. 2018; Yu et al. 2024). The Darongshan Nature Reserve, Yulin City, Guangxi, reported herein, represents the fourth known locality for this species (Fig. 1).

## ﻿Discussion

The conserved external morphology of *Leptobrachella* species makes identification challenging, contributing to the prevalence of cryptic diversity. Integrating molecular phylogenetics and bioacoustic analyses has significantly advanced the taxonomy of this genus (Chen et al. 2024; Yu et al. 2024). Advertisement calls, exhibiting species-specific differences, are a particularly valuable character for delineating *Leptobrachella* species (Köhler et al. 2017; Yu et al. 2024). In this study, the syntopic *L. darongshanensis* sp. nov. and *L. yunkaiensis* possess entirely distinct vocalizations, differing in dominant frequency, call duration, and call interval. Phylogenetic analysis identified *L. darongshanensis* sp. nov. as sister to *L. shiwandashanensis*. However, the profound acoustic divergence—evident in dominant frequency, call duration, and call interval—between *L. darongshanensis* sp. nov. and *L. shiwandashanensis*, coupled with morphological distinctions, provides robust support for recognizing the Darongshan lineage as a distinct new species.

Geographically, the Darongshan Nature Reserve and the type locality of *L. yunkaiensis* (Dawuling Forest Station) both lie within the Yunkai mountain range, separated by a straight-line distance of ca. 120 km. Phylogenetic analysis confirms that the L. cf. yunkaiensis population from Darongshan is closely related to topotypic *L. yunkaiensis*. However, we note that two specimens previously identified as *L. yunkaiensis*—KIZ018211 (from Lidong, Guangxi; GenBank MH055931) and KIZ047782 (from Ehuangzhang Nature Reserve, Guangdong; GenBank MH055932)—exhibit relatively high genetic divergence (3.3–3.9%) from topotypic *L. yunkaiensis* (Suppl. material 2: table S3). This divergence exceeds the typical intraspecific threshold suggested for amphibians (Vences et al. 2005). These specimens were initially identified as *Leptobrachella liui* (Chen et al. 2018) and later assigned to *L. yunkaiensis* based solely on phylogeny without comprehensive morphological re-evaluation (Yu et al. 2024). Therefore, the taxonomic status of specimens KIZ018211 and KIZ047782 requires further investigation incorporating morphological and bioacoustic data to confirm their identity.

The discovery of *L. darongshanensis* sp. nov., currently known only from a small area within the Darongshan Nature Reserve, highlights the ongoing potential for uncovering new amphibian diversity in southern China’s montane regions. Its specialized habitat near headwater streams and limited known distribution warrant attention for conservation assessment.

## Supplementary Material

XML Treatment for Leptobrachella darongshanensis

XML Treatment for Leptobrachella yunkaiensis

## ﻿Additional information

### Use of AI

No use of AI was reported.

### Author ORCIDs

Wei-Cai Chen https://orcid.org/0000-0002-2398-4079

Peng Li https://orcid.org/0000-0001-8311-0544

### Data availability

All of the data that support the findings of this study are available in the main text or Supplementary Information.

## Supplementary materials

Supplementary material 1 Supplementary figure S1 Data type pdf https://binary.pensoft.net/file/1469733This dataset is made available under the Open Database License (http://opendatacommons.org/licenses/odbl/1.0/). The Open Database License (ODbL) is a license agreement intended to allow users to freely share, modify, and use this Dataset while maintaining this same freedom for others, provided that the original source and author(s) are credited.Wei-Cai Chen, Yong-Jian Bei, Peng Li

Supplementary material 2 Supplementary tables S1–S4 Data type xlsx https://binary.pensoft.net/file/1469734This dataset is made available under the Open Database License (http://opendatacommons.org/licenses/odbl/1.0/). The Open Database License (ODbL) is a license agreement intended to allow users to freely share, modify, and use this Dataset while maintaining this same freedom for others, provided that the original source and author(s) are credited.Wei-Cai Chen, Yong-Jian Bei, Peng Li
