# Supplementary figures and images for: ﻿A new species of the genus Leptobrachella (Amphibia, Anura, Megophryidae) from the Darongshan Nature Reserve, Guangxi, China
Source: Zookeys. 2025 Nov 19;1260:171–94. doi: 10.3897/zookeys.1260.161514 (PMC12658442; doi:10.3897/zookeys.1260.161514)

A1

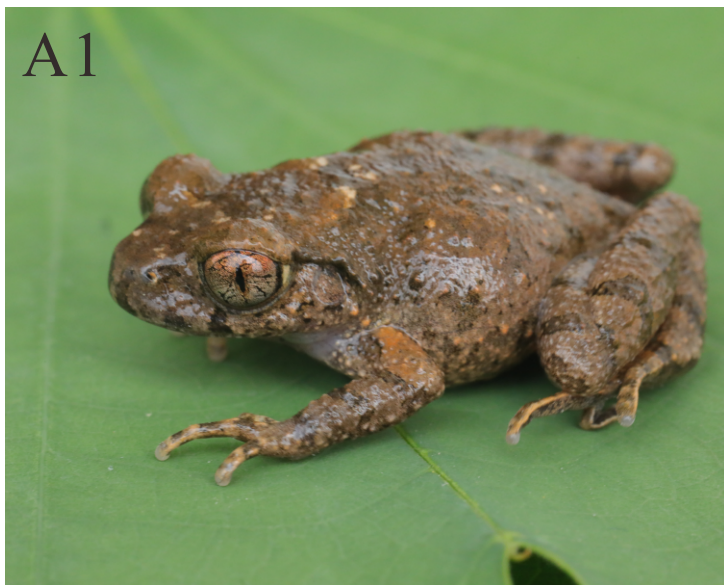

A2

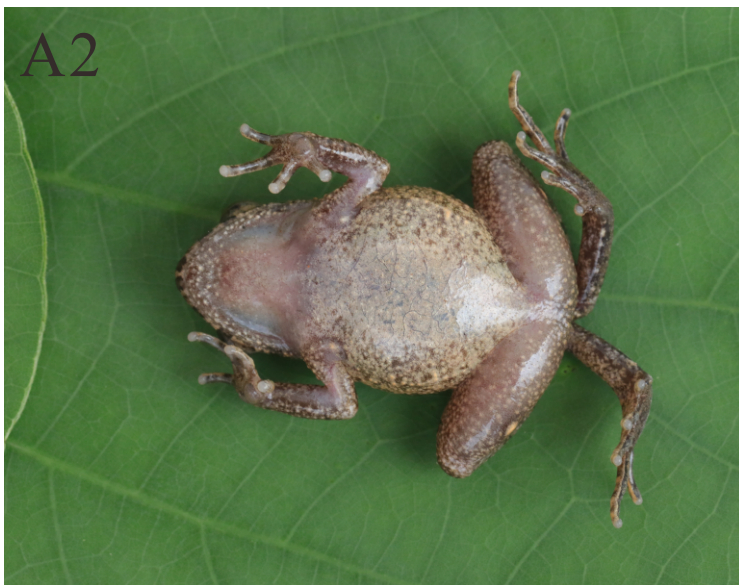

B1

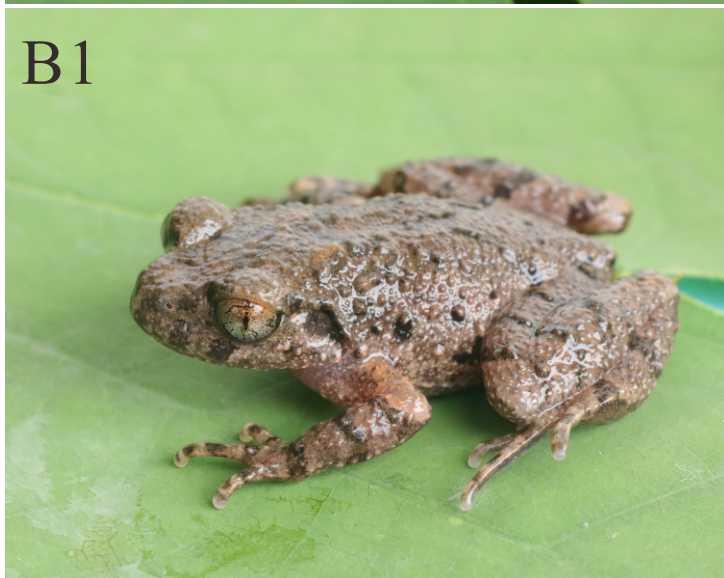

B2

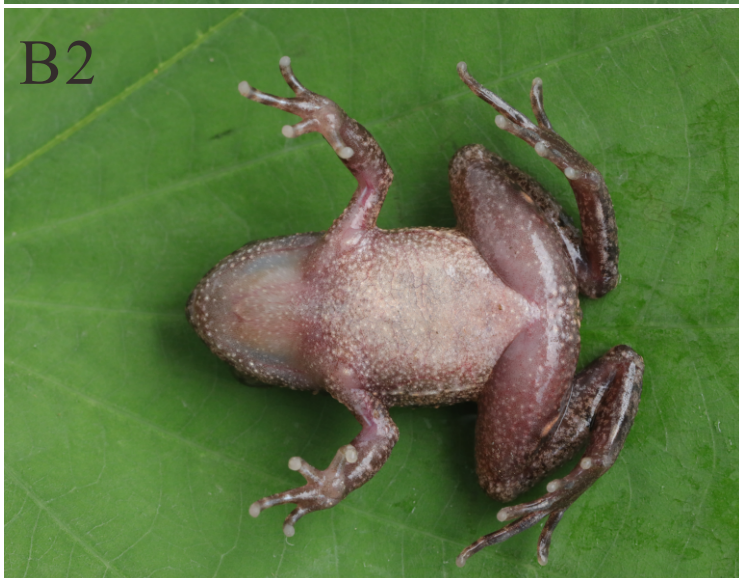

C1

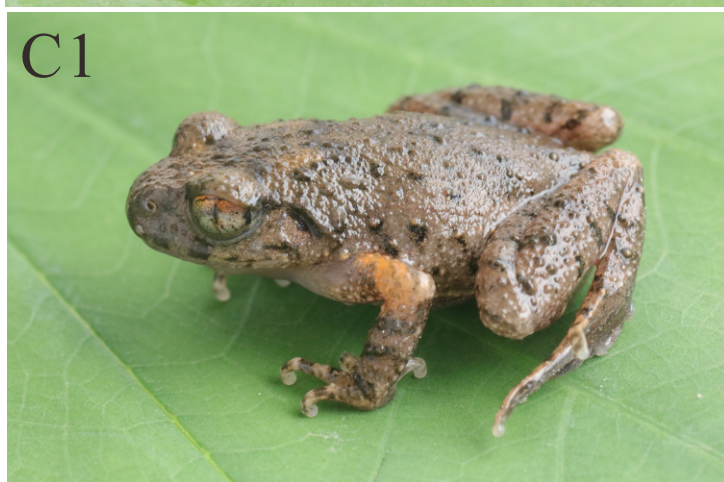

C2

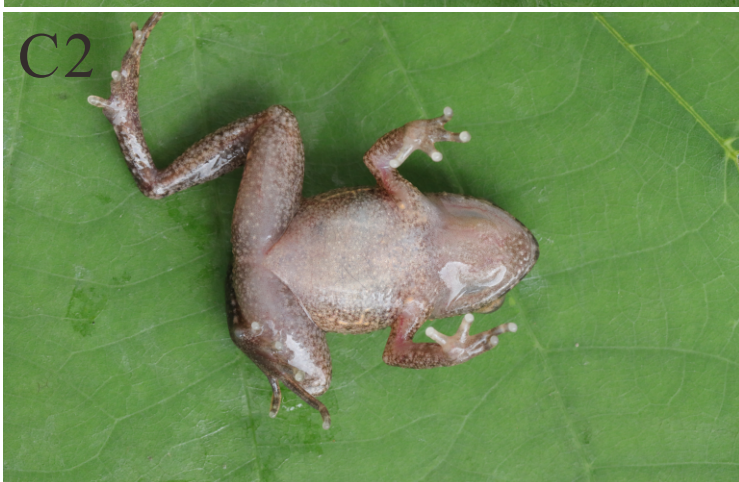

D1

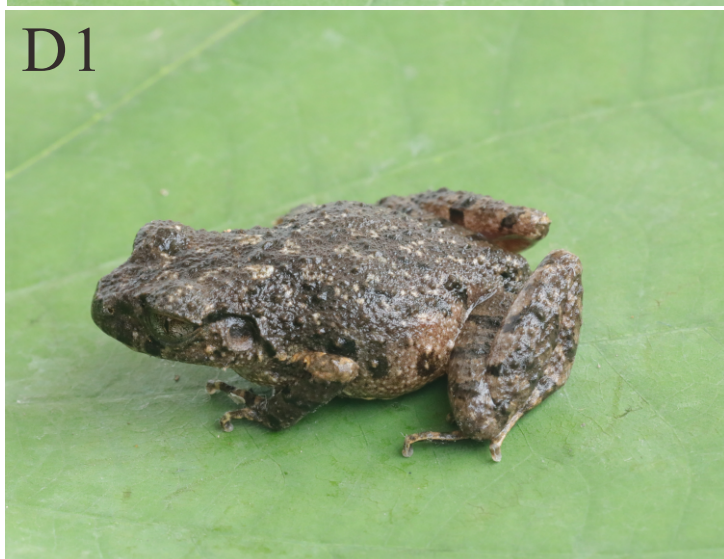

D2

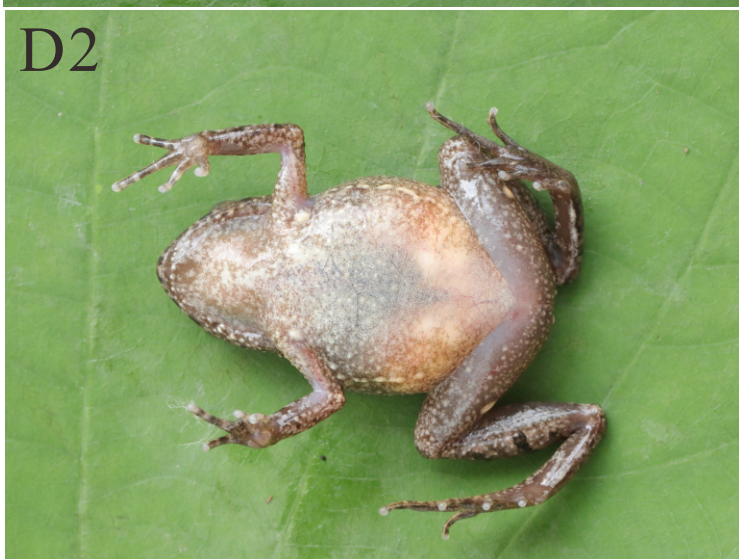

Supplement: Supplementary material 1 — Supplementary figure S1 [file zookeys-1260-171_article-161514__-s001.pdf]
